# The interaction between daylight and fifteenth and sixteenth century glass windows from the Low Countries

**DOI:** 10.1038/s41598-021-00359-7

**Published:** 2021-10-29

**Authors:** Wendy Meulebroeck, Karin Nys, Mathilde Patin, Hugo Thienpont

**Affiliations:** 1 Department of Applied Physics and Photonics, Brussels Photonics, Vrije Universiteit Brussel, Pleinlaan 2, 1050 Brussels, Belgium; 2 MARI Research Group, Department of Art Sciences and Archaeology, Vrije Universiteit Brussel, Pleinlaan 2, 1050 Brussels, Belgium

**Keywords:** Optics and photonics, Applied optics, Optical techniques, Other photonics

## Abstract

The positive impact of daylight on various forms of life is well understood. The daylight conditions a person experiences inside a building strongly depend on the character of the glazing. Contemporary windows maximize the transmission of visible daylight. In post-medieval times glassmakers were confronted with less pure materials. Driven by the Reformation and Counter-Reformation they were at the same time challenged by the demand for increased daylight. Luckily, technological evolutions allowed the production of thinner windows. It is currently an open question if glassmakers in the (Southern) Low Countries during the booming economic period from the fifteenth to seventeenth century made use of the interplay between material and fabrication properties to bring light into the darkness. Therefore, this paper links the impact of glass purity and production technique to light transmission for a well-diagnosed group of excavated glass window pieces from the castle of Middelburg-in-Flanders and a set of roundels, all dating back to between the fifteenth and seventeenth centuries and explores what factors have influenced this technological improvement. A non-destructive approach making use of UV–vis–NIR absorption spectroscopy unveiled that the more recent material is less pure compared to the older dated material but that light transmission was maximized due to the applied production technique.

## Introduction

### The golden age of Flanders and its changing window functions

The economic importance of the Low Countries from the fifteenth to seventeenth century is incontestable. Geographically, the Low Countries are located in Northwestern Europe, forming the coastal lowland region of the Rhine-Meuse-Scheldt delta consisting of the modern Benelux countries, including additional parts of Northern France and Western Germany. Between 1480 and 1560, the most populous cities were located in the counties of Flanders, Brabant and Holland. Cities with access to the sea, such as Bruges in the fifteenth century and Antwerp and Amsterdam in the sixteenth century, experienced flourishing international trade^1^ and a flow of technological knowledge. One example is the production of plain window glass, which in the fourteenth century was monopolized by the glassmaking families of Normandy and Lorraine but diffused towards the Low Countries in the course of the successive centuries. The expanding economy, based on mercantilism, resulted in the emergence of a wealthy middle class, which likewise powered technological innovations. In the case of window glass, the improved Lorraine method allowed the fabrication of larger and more even glass sheets resulting in cheaper windows that could be afforded by others than the wealthy^2^. This gave rise to an increasing popularization of windows in secular buildings from the fourteenth century onwards. These non-figurative windows were constructed by a huge number of small panes joined by lead strips. Various grid patterns were employed over time, including round and diamond (‘lozenge’) shapes typical for the late medieval (fifteenth century), whereas quadrangular shapes were popular during the Renaissance (sixteenth century)^3^. Often, a window was decorated with a so-called stained-roundel, which is an unipartite glass panel rarely exceeding 30 cm in any dimension, whether circular, rectangular or oval, to which vitreous paints were applied and then fired^1^. The sixteenth century was a flourishing period for roundel production in the Low Countries. To meet the high demand, glass-painting ateliers produced the roundels on a semi-industrial scale. This booming roundel business was definitely driven by the Reformation and Counter-Reformation^1,4^. Indeed, the declining importance of the stained, full-colour glazing of churches and other religious buildings forced glass painters to eventually look for different and new customers. Responding to the needs to bring as much light as possible to the interior to help worshippers read the Bible, glass painters turned increasingly to the production of panels painted in a brilliant style^5^. This preference for clear daylight also holds for the non-figurative window fenestration.

### Glass production in late medieval and renaissance northern Europe

Glass production started in the eastern Mediterranean region approximately 2700 years earlier than the glass studied in this paper. The glass industry migrated throughout the Mediterranean towards Europe. Therefore, by the fifteenth century, much was known about the glass-producing technique, which traditionally includes the activities of glassmaking, glass melting and glass forming. Glassmaking is the preparation of molten glass (fritting) from its basic raw materials which are silica (sand) as a network former and alkalis as network modifiers (flux). Between the eighth and tenth centuries, the centralized model of glass production collapsed, and European glassmakers started using locally available sands and plant ashes to produce glass^6^. In contrast to Near and Middle East sands, the sands of many European areas were too impure to be used for glassmaking without prior purification treatments. Oxides of potassium were employed as a principal network modifier to lower the temperature. This is used as a replacement of the oxides of sodium. The raw ingredients had to be chemically balanced because the addition of too much alkali increased the water solubility of the resulting glass. Other oxides such as calcium oxide (lime) acted as counterbalancing stabilizers. Knowledge on these working practices in the early days of glassmaking was obtained in an empirical way, most likely based on trial and error and then documented in glassmaking recipes. The twelfth-century manuscript *Treatise on Diverse Arts* by Theophilus Presbyter^7,8^ is the earliest historic source for medieval and post-medieval glassmaking. Precise instructions on the selection and preparation of the raw materials are given together with a work plan for making crucibles out of clay. Furthermore, it brings in the recommendation to use moderate temperatures and to stir the frit to avoid the forming of lumps.

The alkali source came from the burning of mature trees, including beech and oak, or forest plants such as ferns or bracken^9^. The various melting properties of each alkali-rich material had implications for the temperature and time required to melt the batch. As reported by Jackson and co-workers, glassmakers were skilled and competent artisans who understood their materials. One proof is their knowledge on the preferred use of either beech or bracken ash and sand instead of oak and sand mixtures, allowing glassmakers to work at lower temperatures and requiring them to use less fuel for their production^10^. The applied furnace technology was another factor that strongly impacted the melting conditions^9^. As illustrated in the literature^2,11^, various types of furnaces were employed in the Northern European glass tradition characterized by differences in size, shape and building materials, taking into consideration the essential element of heat sharing between the main and subsidiary furnaces.

### Glass composition in late medieval and renaissance northern Europe

The variation in applied fluxes together with regional differences in soil compositions, resulted in a high variability in major elemental composition. Moreover, it has been proven that glasses produced with ashes, which were harvested at various times throughout the growing season, differed in composition^12^. Wood/plant ash glasses are typically subdivided into potash and high-lime-low-alkali (HLLA) glasses based upon their K_2_O/CaO ratio^13^. Researching the relation between glass type and chronology has shown that fifteenth-century material tends to be potash glass, whereas glass from sixteenth and seventeenth contexts often has an HLLA glass signature. However, an overlap exists between the uses of both glass groups for the early periods. According to the literature, the time transition point from potash towards HLLA glass strongly depends on the region. HLLA glass was identified by Wedepohl in German sites from fourteenth to seventeenth-century contexts^13,14^. From the second half of the fifteenth century to the end of the seventeenth century, both potash glass and HLLA are present in Northern France^15^. Initially, potash glass was mainly appearing in the northwestern region, and HLLA glasses were appearing in the northeastern region close to the German borders. Afterwards, the diffusion of HLLA glasses from east to west was observed^15^. For England, the research of Dungworth and co-workers demonstrated the occurrence of HLLA glasses from the second half of the sixteenth century onwards^16^. References are scarce for the Low Countries. The existing literature suggests that most of the non-figurative fifteenth- to seventeenth-century material is HLLA^3^ and that the composition of the roundel glass of the Low Countries does not show significant difference from ordinary window glass, with HLLA glass being the dominant glass type^17^.

### Unravelling the interplay between glass technology and daylight transmission

For the non-figurative windows, the small size of the panes and the lead cames contributed to a window that would transmit much less light than our modern windows^16^. Another aspect that influenced the light transmission was the non-whitish tint of the glass originating from the applied raw materials. Naturally coloured glasses mainly owed their colour to iron impurities, which were present in the raw materials. In most silicate glasses, the two oxidation states Fe^2+^ and Fe^3+^ simultaneously occurred. The iron redox ratio (Fe^2+^/Fe^3+^) was influenced by the used raw materials^18^. Apart from the purity of the raw materials, the glass-forming technique could have also played an important role. Craftsmen could, for example, have tuned the glass thickness with the purity of the raw materials. Since Roman times, glassmakers were applying spinning techniques to produce window glass using either the crown or the cylinder glass technique. To obtain crown glass, a glass was blown as a bubble that was then opened and spun around to form a disk of glass. The cylinder glass technique consists of blowing a glass bubble that is elongated to form a cylinder, which is then cut open and flattened^2^. Until the last quarter of the sixteenth century, two production centres dominated the glass market: French (Normandy) crown- and the Lorraine (Rhine area) cylinder-production centres. In the fourteenth century, improvements in the cylinder technique, which probably first took place in Bohemia, resulted in an increased flatness of the window surface. This knowledge was taken to Lorraine^2^. In the second half of the seventeenth century, migrations of glass workers took place due to economic and political reasons, resulting in the diffusion of crown- and cylinder glass-production centres located in the Low Countries, England, Germany, France and so on^16,17^.

The principal goal of this paper is to investigate if a chronological evolution exists in the amount of light that was transmitted through non-figurative windows. Starting from two selected subsets of window fragments that are attributed to the fifteenth and sixteenth centuries, respectively, we first study the glass purity and bring this into relation with the glass thickness, after which we compare the calculated transparency values. As a subsequent question, we will research if the glass sheets were imported or were produced locally. We also have the idea to explore if one was preferred over the other in view of the purity of the materials. In a final step, we will compare the purity and transparency properties of a selection of roundels, with known dating and all originating from the Low Countries, with those of the non-figurative windows. Most of the investigations were carried out with Ultraviolet–visible–Near-InfraRed (UV–vis–NIR) absorption spectroscopy because our research group has proven that this non-destructive technique is especially suitable to make a first-line classification of glass fragments based on their iron ion concentration levels^19,20^. In doing so, we also fulfilled the requirement of avoiding the use of destructive material analysis techniques on the full collection. This methodology is possible thanks to modern technological developments that led to the availability of handheld X-Ray-Fluorescence equipment^21,22^, mobile Raman instruments^23,24^ and portable spectrometers^25,26^ allowing a non-destructive material analysis. Chemical analyses made on a subset of 11 sampled pieces of the non-figurative window collection, allowed us to define the major elemental composition.

## Materials

The first case study concerns a collection of 29 non-figurative window glass fragments that were excavated on the site of the castle of Middelburg-in-Flanders (Maldegem, East Flanders province, Belgium). The castle was founded during the mid-fifteenth century by Pieter Bladelin, who was a counsellor of the Dukes of Burgundy. It had fallen into ruins by the beginning of the eighteenth century^27^. This sample set was selected because earlier research has shown that the samples can be classified into two distinct groups all linked to specific aspects that are relevant for our research^27^. A first group contains five fifteenth-century lozenge-shaped fragments produced with the crown technique. The second group (24 fragments) contains 16th-century material fabricated with the cylinder-production technique characterized by the quadrangular pattern shape. We abbreviated these types in the figures (captions) and when reporting quantitative data as ‘L.’ and ‘Q.’, respectively.

For the second case study, we selected six roundels whose dates span the centuries of interest starting in the first half of the sixteenth century (IA552 and AV1172) and proceeding towards the mid- (IA4019) and second half (AV1172) of the sixteenth century and ending in the seventeenth century (IA4039 and AV8097). All panels originate from the (Southern) Low Countries and are linked to either the modern Antwerp (AV1171 and AV1172), Brussels (IA552) or Leuven (IA4019 and IA4039) region. Panel AV8097 could not be linked to a specific region based on its stylistic properties. The literature presents pictures and descriptions for IA552^28^, AV1171^29^ , AV1172^29^, IA4019^28^ and AV8097^29^. Roundel IA4039 is attributed to the famous 17th-century glass painter ‘Jan de Caumont’ from Leuven.

## Results and discussion

### The sixteenth-century non-figurative windows transmit more light compared to their fifteenth-century counterparts, despite the less pure character of the applied materials

**Figure 1:**
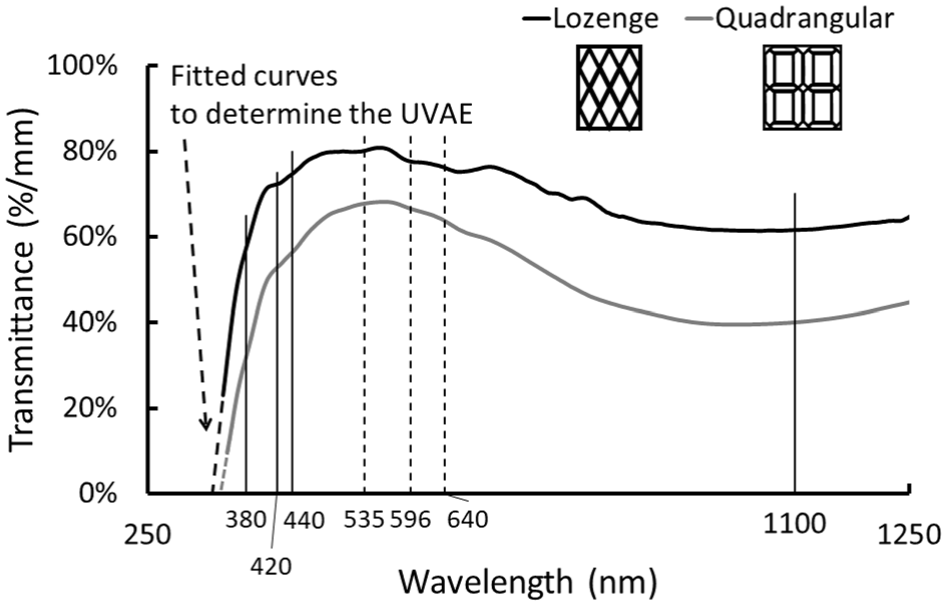
Transmission spectra normalized to a 1-mm glass thickness show the presence of iron and cobalt impurities. Solid (Fe^2+^ and Fe^3+^) and dashed (Co^2+^) lines mark the absorption band positions. The differences in UVAE values are visualized.

To investigate the glass-transmission properties, we started from the recorded optical transmission spectra. Transition metal ions absorb light in the UV–vis–NIR region of the electromagnetic spectrum at fingerprinting wavelengths that have been frequently reported in the literature^18^. Earlier research of our group has shown that, for naturally coloured plain-glass fragments, the absorption bands of iron,—in particular, but often also of cobalt—are observable in the recorded spectra^30^. Therefore, iron and cobalt are the main drivers hampering light transmission through the windows. The shapes of the recorded spectra indeed demonstrate the presence of both iron ion types Fe^2+^ and Fe^3+^ (Fig. 1). The presence of divalent iron is designated by a characteristic absorption in the NIR region at 1100 nm^18,31^, whereas the trivalent iron bands are situated in the UV region of the spectrum with distinct bands close to 380, 420 and 440 nm. Figure 1 points out the positions of the iron-absorption bands using solid lines. A clear difference in UV and NIR absorption is observed between the lozenge and the quadrangular group, indicating a difference in Fe^2+^ and Fe^3+^ impurity levels (Supplementary Table S1). In addition to iron, cobalt impurities are also present. Cobalt is characterized by the presence of three successive absorption bands close to 535 nm, 596 nm and 640 nm (for soda-rich glass) attributed to the Jahn–Teller split transition A2 → T1(P) of Co^2+^^18^. The positions of these bands are plotted as dashed lines in Fig. 1. For all 29 fragments, these cobalt bands are observed. The weak presence of the peaks, as well as the knowledge that very small concentrations as low as 50 ppm for a 2-mm-thick sample can be observed in optical spectra^32^, allow us to conclude that cobalt is present at the impurity level but in much lower concentrations compared to the iron-impurity levels. In relation to iron, cobalt has stronger light-absorption properties, which are caused by its higher extinction coefficient^18^. Chemical analysis made on a subset of 11 sampled pieces (Supplementary Table S2) confirmed the difference in iron (ΣFe = Fe^2+^ + Fe^3+^) concentrations. Compared to the lozenge glass, the quadrangular group is characterized by higher ΣFe concentrations (1.1 wt.% ± 0.04 versus 0.6 wt.% ± 0.05)^30^. The cobalt concentrations were below the detection limit of the applied technique.

A first optical measure that will be employed to ‘quantify’ the purity of the 29 non-figurative window samples is the glass colour. When the sample is purer, its colour values are more closely positioned to the white point of the colour diagram. Apart from the purity of the sample, the thickness also affects the colour. We therefore need to calculate the so-called intrinsic colour, which is the colour with the sample thickness normalized to 1 mm. Important to remark is that we use the thickness at the glass material position where the light transmission was measured. It goes without saying that, at this location, all deterioration must be removed prior to measurement. For the hereto reported set of samples, Hilde Wouters performed this pre-processing step. Being a glass conservator, she had the necessary expertise and tools to clean a measurement region of approximately 12 mm^2^.

The calculated colour values are supplemented with two additional optical parameters. This is needed because care needs to be taken when trying to map intrinsic glass colour to glass purity because a manipulation of the redox condition can lead to various colours starting from the same glass composition. Therefore, when studying iron impurities in naturally coloured glass, a combined study of the intrinsic colour with the UV-absorption edge (UVAE) values and the absorbance at 1100 nm is advised^20^. This absorbance value is directly proportional to the Fe^2+^ concentration^18,31^. Charge transfer absorptions between oxygen and trivalent iron ions cause glass absorption in the UV region. At higher Fe^3+^ concentrations, the UVAE shifts to longer wavelengths. Other factors influencing the position of the UVAE are the presence of other ions, such as Fe^2+^, Ti^4+^ and Mn^2+^, the alkali content and the thermal history of the glass fragment^33^. Therefore, the UVAE is only an indicative parameter for Fe^3+^ glass impurities. Recent research of our group has demonstrated the need to deviate from the standard UVAE definition applied for modern glasses when one is dealing with ancient glasses^20^. For ancient glasses, the UVAE must be obtained by fitting the transmission spectrum for 1-mm material with a linear curve in the ultraviolet spectral region and by determining the wavelength at which the fitted line goes to zero. As an illustration, we have plotted in Fig. 1 the linear curves that we fitted to the two showing light-transmission curves.

**Figure 2:**
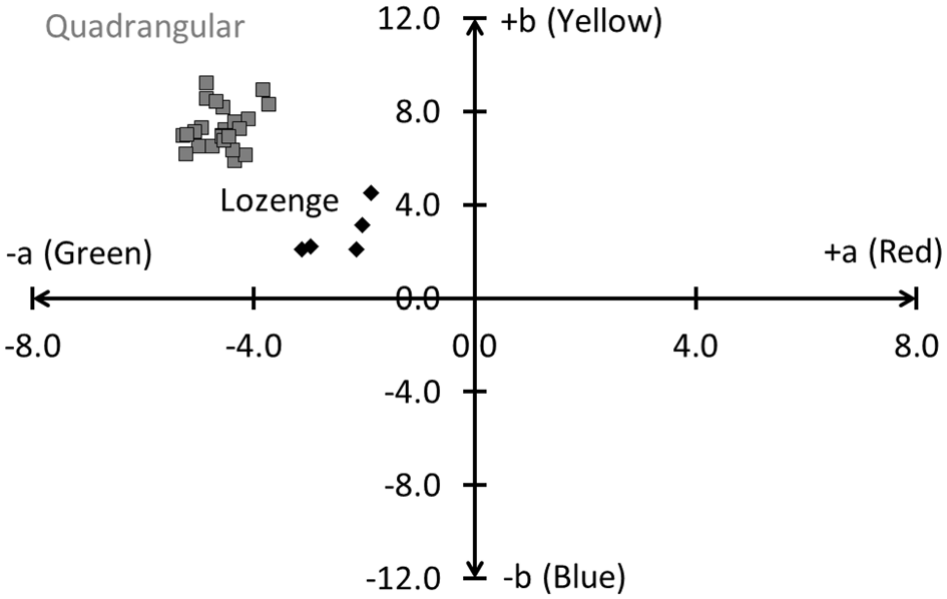
The paler appearance and purer character of the lozenge glass (filled diamond) compared to the quadrangular glass (filled square) is proven by its CIELab colour values, which are closer positioned to the origin of the axes.

**Figure 3:**
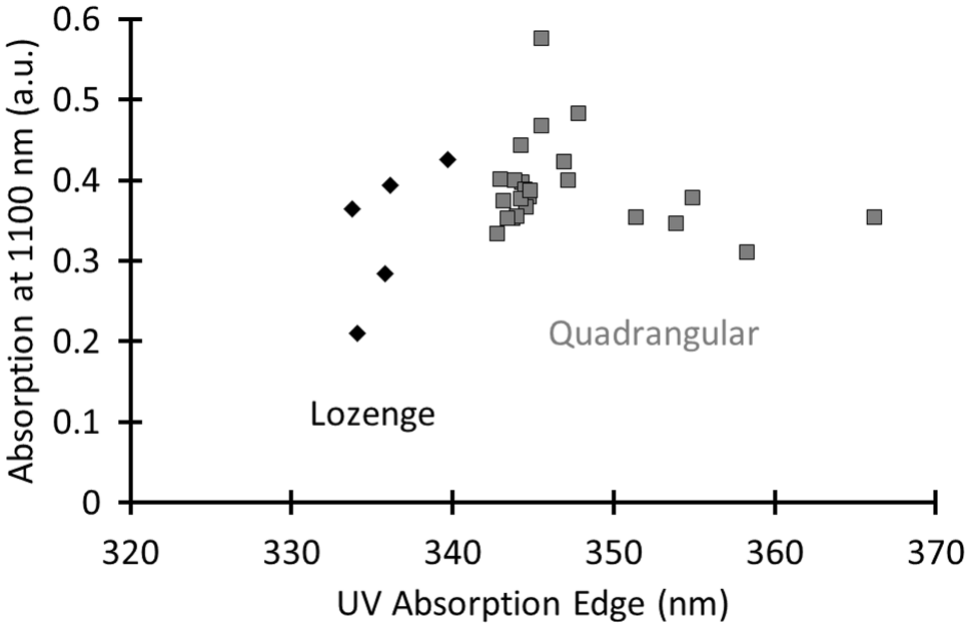
A clear difference in the UV-absorption edge value is visible between the lozenge (filled diamond) and the quadrangular glasses (filled square), confirming a difference in impurity levels between both glass types.

The calculated colour coordinates (Supplementary Table S1) show that the lozenge glass has a paler hue compared to the more greenish quadrangular glass, with colour values being more closely positioned to the origin of the axes on the CIELab colour system (Fig. 2). On the abscissa, the values of a indicate green to red colours, whereas b changes from blue to yellow. The origin of the axis indicates the absence of colour. We selected this colour space because the same amount of numerical change in CIELab values corresponds to roughly the same amount of visually perceived change^34^. As can be seen in Fig. 2, all samples cluster in two well-separated groups (L.: a −2.4 ± 0.6 and b 2.8 ± 1.1; Q.: a −4.6 ± 0.4 and b 7.3 ± 0.9). This classification is mainly due to a difference in absorption in the UV region (Fig. 3). The obtained UVAE values for the lozenge group range between 333 and 340 nm (335.9 ± 2.3), whereas the UVAE values for the quadrangular group are situated between 342 and 366 nm (347.2 ± 6.1).

Next, we quantified the colour difference a glass maker would have observed between two 1-mm-thick glass samples with glass purities respectively equal to those of the lozenge and the quadrangular group. Starting from the two colours in the CIELAB space, which are the average intrinsic colour values for the two glass types, we calculated the colour difference ΔE_ab_ using the CIE76 formula^34^. Whereas a ΔE_ab_ value of ~ 2.3 corresponds to a just noticeable difference by a standard human eye, the colour difference we observed is 6.4 ± 0.84. The difference in glass purity is thus distinguishable to the eye.

The observed dissimilarity in purity between the two groups indicates that various glass recipes and/or raw materials were employed, leading to different furnace conditions. Combining the optical and chemical data^19^, we estimated the Fe^2+^/ΣFe ratio (Supplementary Table S1) and concluded that both iron ion types are present in the two groups with Fe^2+^/ΣFe values ranging between 0.37 and 0.55 wt.% for the lozenge group, as well as between 0.27 and 0.34 wt.% for the quadrangular group, indicating that the quadrangular group is slightly more oxidized compared to the lozenge group. The latter is confirmed by the longer UVAE values for the quadrangular group^30^. We remark that the lozenge samples have higher MnO concentrations (0.7 wt.% ± 0.1 versus 0.5 wt.% ± 0.01), which might indicate a deliberate addition of manganese to counteract the natural colour of iron. Moreover, we note that the overall alkali concentrations for both groups are comparable (wt.% [Na_2_O + K_2_O] equal to 7.8 ± 0.3 and 7.8 ± 0.9 for Q. and L., respectively) and as such have no additional influence in augmenting oxidizing conditions^30^.

In a next step, we related our observations on the glass purity, with the glass thicknesses measured during the macroscopic research. It was reported that both identified groups not only differ in shape but also in thickness^27^. The lozenge material is at least 3 mm thick, whereas the quadrangular material features a thicknesses of up to 2 mm. These observations point to an inverse relationship between glass purity and glass thickness.

**Figure 4:**
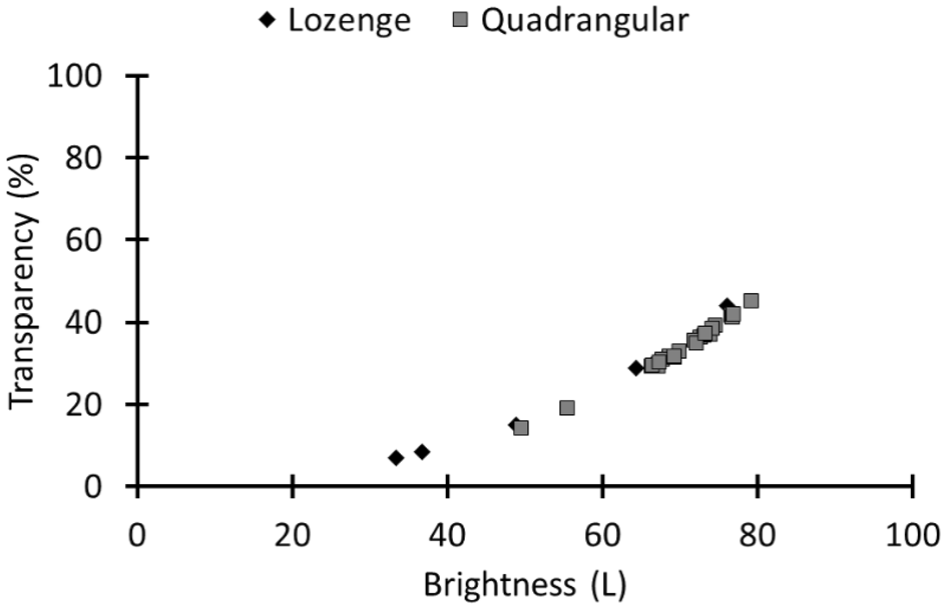
The transparency and brightness (L colour coordinate) values calculated from the simulated light transmission spectra with fixed thickness (3 mm for lozenge and 2 mm for quadrangular) show a higher light transmission for the later-dated quadrangular window glass.

From the previous paragraphs, we know that, on the one hand, a difference in purity exists between both types of fenestration. On the other hand, we are also aware of an occurring difference in material thickness. Therefore, the question arose if there is a noticeable difference in transparency between the two types of windows. The transparency is calculated from the transmission spectra (Supplementary Table S3). A fully transparent window characterized by a 100% transmission over the entire visible wavelength range (380–780 nm) has a transparency value of 100%. The calculation was performed on simulated spectra starting from the intrinsic spectra. As we explained before, the high level of material deterioration required a cleaning of the material prior to the optical recording. As simulated thicknesses, we applied the boundary values obtained from the macroscopic research^27^ (i.e. 3 mm for the lozenge glass and 2 mm for the quadrangular glass). Transparency values are linked to the L colour parameter, which quantifies the brightness of the transmitted light. Therefore, we calculated both the transparency and the brightness values. Figure 4 shows the results, which demonstrate that, despite the greener tint of the quadrangular material, this window type transmitted more daylight compared to the lozenge windows due to its lesser thickness (L.: 21% ± 16% of transparency; Q.: 34% ± 7%). The large variations on the calculated values are induced by the more severe weathering conditions of the lozenge material, inducing larger errors on the measured sample thicknesses and, as such, on the derived intrinsic transmission spectra.

### The raw materials most probably originated from local workshops

Further study of the cobalt band positions provided the first hints of a calco-potassic composition. Earlier research showed that the ligand field strength influences the positions of the absorption peaks, and therefore, a change in chemical composition results in a shift of the bands. Pieces of glass from the calco-calcic glass family have their first cobalt absorption band located at shorter wavelengths (~ 526.5 nm ± 1.5 nm) compared with soda-based glass compositions, where the band is shifted towards longer wavelengths (535 nm ± 2 nm)^35^. Starting from the chemical composition and following the schemes available in literature^3,36^, both glass pattern shapes were assigned to different HLLA subgroups (Supplementary Table S2). All glass pieces have CaO/(CaO + K_2_O) greater than about 0.75 (0.78 ± 0.05 and 0.76 ± 0.05 for Q. and L., respectively) with less than about 10% alkali (Na_2_O + K_2_O). Indeed, subgroups are often easily defined due to the inhomogeneity of post-medieval and Renaissance glass pieces. Examples of HLLA subclassifications are diverse in the literature, including the HLLA(I-III) groups distinguished at a Polish excavation site and the HLLA(A-E) groups defined during the study of Belgian non-figurative window fragments^3,37^.

In what follows, we analyse and discuss the differences in applied glass technology and in the geographical locations of the glass production centres. First, we compare the chemical data of the major elements (Supplementary Table S2 and reference^30^), with published data (Table 1) on regional specific potash-lime glass compositions from the broad region neighbouring Middelburg-in-Flanders. Making a comparison with the Belgium region was possible in this study due to the work of Schalm, who studied a set of 434 window glass fragments from 12 sites in Belgium^3^. In this paper, we focus on his ‘Group B’ because of the reported fifteenth to seventeenth-century dating. This group contained 59 samples. For the French glass composition, we refer to the study of Barrera and Velde on 11 individual sites^15^. In their paper, the potash-lime groups are indicated as Type A with respective subgroups III (second half of fifteenth to the end of the sixteenth century) and IV (end of 16th to the end of the seventeenth century). A group of 30 samples originating from four German sites define the wood ash-lime group that Wedepohl described^14^. Finally, for post-medieval England, we refer to the HLLA 2a group (c. 1567–c. 1600) that Dungworth identified^16^. Here, the study encompasses samples originating from several sites scattered throughout the broad English region.

**Table 1:** Comparison between glass identified in this work and fifteenth to seventeenth c. material from Belgium^3^, fifteenth to seventeenth c. French material^15^, fifteenth to seventeenth c. wood ash lime glass from Germany^14^, and British sixteenth to seventeenth c. material^16^.

	Belgium	Belgium	Belgium	France	France	Germany	UK
	This work L	This work Q	Group B (Schalm)	Type A III (Barrera & Velde)	Type A IV (Barrera & Velde)	Wood ash lime (Wedepohl)	HLLA 2a (Dungworth)
	15th. c	16th. c	15th–17th c	15th–16th c	16th–17th c	1400–1600 AC	c. 1567–c. 1600
SiO_2_	60.4 ± 7	60.5 ± 0.7	56 ± 2			55.86 ± 3.82	60.4 ± 1.8
Al_2_O_3_	3.8 ± 0.6	3.7 ± 0.1	3.7 ± 0.8			2.50 ± 0.87	2.8 ± 1.0
Fe_2_O_3_	0.6 ± 0.1	1.1 ± 0.1	1.1 ± 0.4			0.82 ± 0.35	1.01 ± 0.20
MnO	0.7 ± 0.1	0.52 ± 0.01	1.2 ± 0.6			1.28 ± 0.71	0.94 ± 0.37
Na_2_O	1.4 ± 0.5	2.0 ± 0.1	1.0 ± 0.6	0.1–0.3	0.3–0.4	2.58 ± 0.67	1.4 ± 0.7
K_2_O	6.4 ± 1.4	5.8 ± 0.3	7.1 ± 0.9			5.10 ± 1.53	5.6 ± 1.6
MgO	2.9 ± 0.4	2.8 ± 0.1	3.7 ± 0.3	1.8–2.9	2.1–2.6	4.06 ± 0.58	3.4 ± 0.5
CaO	20.4 ± 0.7	20.8 ± 0.4	23 ± 1			23.69 ± 2.23	21.5 ± 1.9
K_2_O/CaO	0.3 ± 0.08	0.3 ± 0.01	0.3 ± 0.1	0.1–0.4	0.1–0.25	0.22 ± 0.08	0.3 ± 0.1
Cl	1.1 ± 0.5	1.0 ± 0.1	0.2 ± 0.1			0.40 ± 0.16	0.3 ± 0.2
P_2_O_5_	1.6 ± 0.3	1.3 ± 0.1	2.7 ± 0.8			3.34 ± 0.82	2.1 ± 0.2

**Figure 5:**
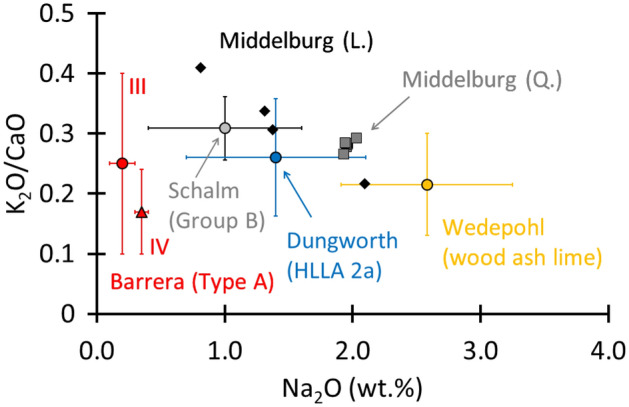
Plotting the Na_2_O concentration against the K_2_O/CaO ratio shows that all groups have comparable K_2_O/CaO ratios but deviating soda levels. Considering all groups with a fifteenth century assignment shows that the lozenge group (filled diamond) best matches Group B of Schalm. The same exercise used for the quadrangular group (filled square), unveils some overlapping behaviour with the German wood ash lime that Wedepohl researched, as well as the British HLLA 2a material that Dungworth described.

**Figure 6:**
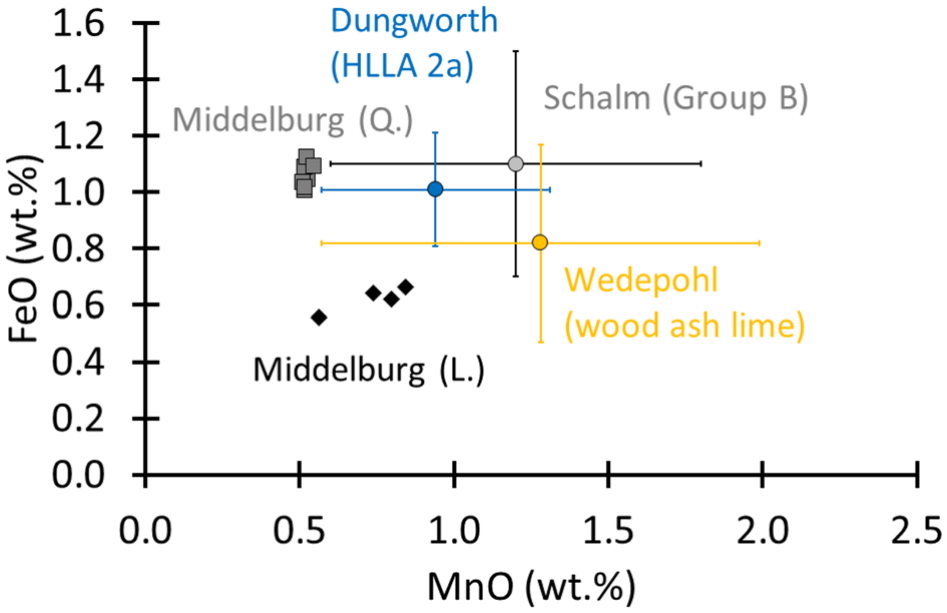
The Mn-Fe chart shows that the lozenge material (filled diamond) has lower iron levels compared with the Group B material that Schalm reported. Meanwhile, the quadrangular material (filled square) unveils iron and manganese levels which are close to those of the Group B material of Schalm and the HLLA 2a group of Dungworth.

Starting from the reported dating of the different considered groups, we selected the following material for comparison with the fifteenth century dated lozenge material: Group B with Belgian material (fifteenth to seventeenth century), Type A III group with French material (fifteenth to sixteenth century) and the wood ash lime group containing German compositions (1400–1600 AC). The soda levels of the lozenge group (1.4 ± 0.5 wt.%) are in the range of those reported for the Belgian HLLA Group B material (1.0 ± 0.6 wt.%). The French Type A III samples provide the compositions with the lowest amount of soda (0.1–0.3 wt.%), whereas the German material is on average characterized by higher soda levels (2.58 ± 0.67 wt.%). The K_2_O/CaO contents of all groups are in the same range with average values close to 0.3 wt.% (Fig. 5). The same observation is true for the MgO levels (L.: 2.9 ± 0.4 wt.%, Belgium Group B: 3.7 ± 0.3 wt.%, French Type A III: 1.8–2.9 wt.% and German wood ash lime: 4.06 ± 0.58 wt.%). A final aspect that discerns the lozenge group samples is the observation of a lower iron concentration (0.6 ± 0.1 wt.%) in comparison with the other groups (Belgium Group B: 1.1 ± 0.4 wt.% and German wood ash lime: 0.82 ± 0.35 wt.%). All groups show manganese concentrations in the same order of magnitude (Fig. 6).

For the quadrangular material, we performed the comparison of the fifteenth to seventeenth centuries material Group B with Belgian material supplemented with the sixteenth to seventeenth centuries Type A IV material of France, the wood ash lime group with German material and the HLLA 2a group (c. 1567–c. 1600) identified for the British region. As with the lozenge material, a similarity in soda levels is also observed here between the Middelburg-in-Flanders material (2.0 ± 0.1 wt.%) and the comparative Belgian Group B material (1.0 ± 0.6 wt.%). Also, the British group HLLA 2a material (1.4 ± 0.7 wt.%) and the wood ash lime group from Germany (2.58 ± 0.7 wt.%) show soda levels that are within the error margins. These soda concentrations are much higher than that of the French Type A IV material (0.3–0.4). As with the lozenge group material, here, all considered groups are also characterized by comparable K_2_O/CaO ratios (close to 0.3 wt.%) (Fig. 5) and MgO contents (close to 3 wt.%). The Mn-Fe chart (Fig. 6) shows that the quadrangular material has manganese levels which are close but slightly lower (0.52 ± 0.01 wt.%) compared with the Belgian (1.2 ± 0.6 wt.%), German (1.28 ± 0.71 wt.%) and British (0.94 ± 0.37 wt.%) materials. The iron levels are all within the same range, approximately 1 wt.%.

The above-described findings might favour the identification of local production. Knowing the typical high variability in major elemental compositions due to regional differences in soil compositions and/or applied ashes, we believe that both the lozenge and the quadrangular material from Middelburg were produced in the broad area of the Low Countries. The difference in iron concentrations between the Middelburg samples and Group B that Schalm reported, might have stemmed from the use of a different sand source, whether or not a potential difference in the fluxing agent was also found. It is reported that seaweed ash leads to lower iron content than beech wood glass pieces do^36^. For the lozenge material, the strong correlation (R^2^ = 0.99) between sodium and chlorine is apparent.

From the above explanations, we conclude that both glass pattern shapes were fabricated with different recipes. This might not be entirely unexpected because for stained glass, the use of local materials appeared to be a common practice during this period. Illustrations that support this statement are the recommendations that the Antwerp Guilds made to use local glass^17^, as well as the Raman signal variability that was observed for stained glass pieces originating from different locations^38^. On the one hand, the lozenge and the quadrangular glass could originate from two different local glass workshops. In this case, the lozenge glass-production centre was likely closer to the seaside, and the quadrangular material was produced closer to the eastern borders. The first factor that supports this statement is the lower iron content potentially originating from seaweed ash for the lozenge glass. The strong correlation between sodium and chlorine might also indicate that the glassmakers collected their alkaline raw materials near salty seashores. Second, the seaside region is geographically closer to the French region. The fact that the lozenge material was fabricated using the crown-glass production method highlights the knowledge transfer that occurred from France towards other European regions, including the Low Countries. It is an open question if the historically documented diffusion of French crown glassworkers arriving in England in 1567 occurred through the Low Countries or if the diffusion to both regions occurred independently. Finally, we remark the compositional similarities of the Middelburg material with the average compositions of 2a group historic window glass pieces from England that Dungworth reported. This supports the existing trade between mainland Europe and English glass producers^16^. On the other hand, both glasses could have been produced at the same workshop, and glass workers at a certain moment in time may have noticed that the cylinder method was preferential in view of a higher light transmission, even if less pure raw materials were employed. The latter might then explain the difference in the raw materials. The information that window glass manufactures were taxed on the weight of the materials would obviously also have played an import role in the preferred use of the quadrangular material leading to thinner glass sheets^16^.

Independently of which of these two scenarios is the valid one, it is clear that fifteenth to sixteenth-century glassmakers in the Low Countries were using technological innovations to maximize daylight transmission. The researched colour difference ΔE_ab_ value of 6.4 between the two glass groups proves that the colour difference was definitely notable to the glassmaker, and preference was given to maximize daylight upon the ‘whiteness/colour’ of the windows.

### Compared with the non-figurative window glazing, roundels transmitted more daylight. The purer the roundel material, the more that light was transmitted

**Figure 7:**
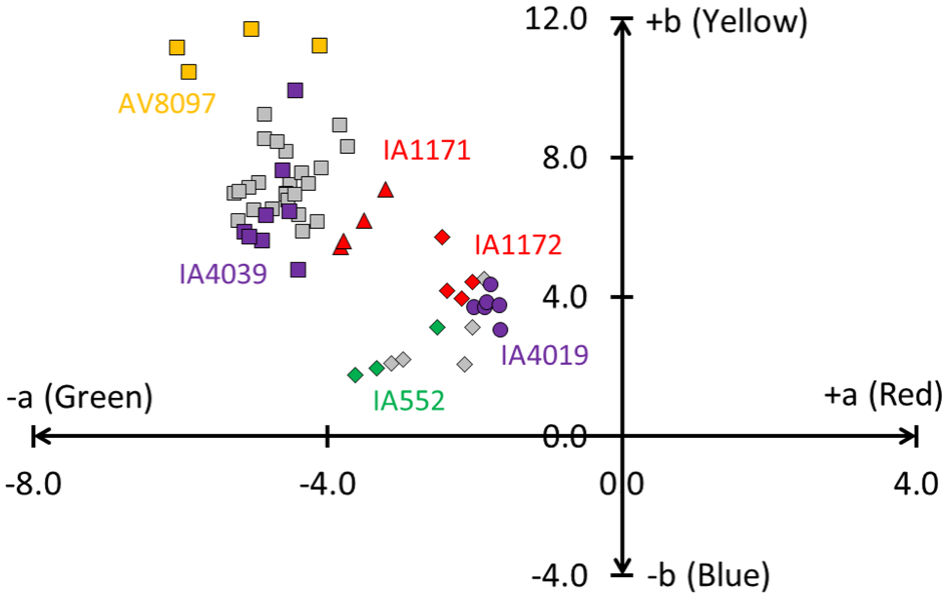
The colour values of the roundels show that the earlier roundel material (IA552, IA1172 and IA4019) is purer compared with the later material (IA4039 and AV8097). The following symbol code was applied: early 16th c. (diamond), mid-16th c. (circle), late 16th c. (triangle), and 17th c. (square). The colour filling of the symbols represent the location of origin: Antwerp (red), Brussels (green), Leuven (purple) and undefined (orange). The colour coordinates of the Middelburg material are plotted in grey.

**Figure 8:**
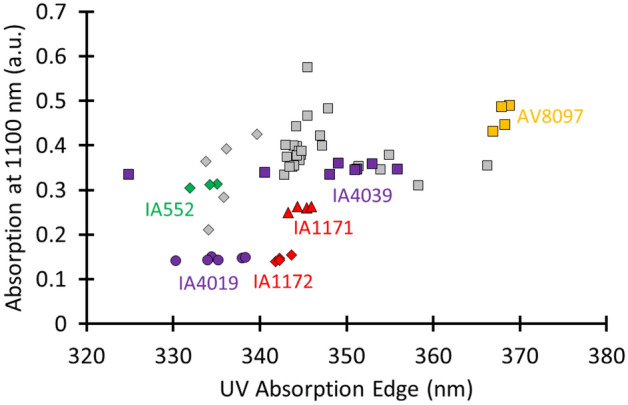
Plotting the UVAE values against the absorption values at 1100 nm confirms the purer character of the earlier material. The same legend code as in Fig. 7 was applied.

Because we were not permitted to sample the material, the purity was studied based on the earlier described optical parameters. Roundels are typically characterized by several areas with painted silver stain or grisaille layers. Therefore, it was important to first identify those locations where no (or a minimum amount of) paint layer was present. Only for roundel IA4039 did it appear that the entire surface was coated with a thin grisaille layer. An analysis of the optical parameters demonstrated that a parallel could be drawn between the purity of the non-figurative windows and the roundels. For example, the earlier material (IA552, IA1172 and IA4019) is purer compared with the later material (IA4039 and AV8097) (Supplementary Table S4). In comparison with the older dated roundels, the colour coordinates of roundels IA4039 and AV8097 are further away from the origin of the Lab colour diagram (Fig. 7), and their UVAE values are also higher (Fig. 8). The deviant purities of roundels IA4019 and IA4039, which both originate from the same region (Leuven), prove that the difference in glass purity is not caused by the geographical location of the workshop but rather to a chronological difference in applied production technique. At first sight, roundel IA1171 appears to be an outlier. Based on its stylistic properties, this roundel was classified in the second half of the sixteenth century, while its glass purity matches with that of the later non-figurative window material. It is an open question if this roundel was painted on a bare glass sheet that was still in stock from an earlier date or if the glass purity transition point for roundel material must be situated at the end of the sixteenth century. Another observation is that every optical parameter, measured at different locations within one roundel, leads to a specific cluster and that the different clusters do not overlap. Another remarkable notice is that for each of the earlier defined non-figurative window groups, a roundel was found for which the colour values, the absorption at 1100 nm and the UVAE values, overlap. These are roundel IA552 for the lozenge group and roundel IA4039 for the quadrangular group.

**Figure 9:**
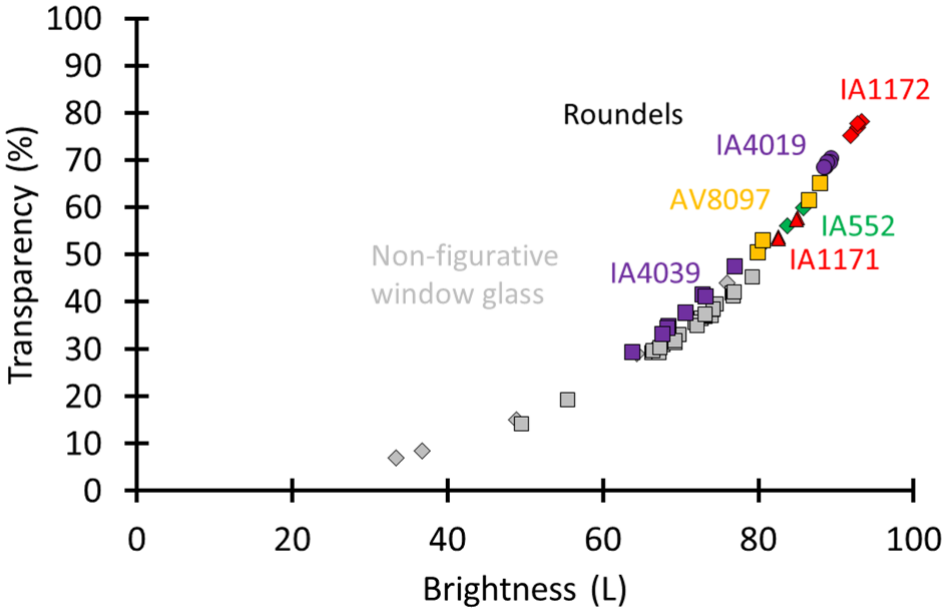
The brightness and transparency values show that more daylight was transmitted through a roundel compared with the non-figurative window glazing. The same legend code as in Fig. 7 was applied.

Finally, we studied the daylight transmission of these roundels (Fig. 9) and concluded that more daylight was transmitted through the roundels compared with the non-figurative window glazing. This is with the exception of roundel IA4039. The higher absorption might stem from the grisaille layer. The higher light transmission of the roundels might reflect their social status (i.e., roundels were considered to be luxurious materials). A second conclusion is that in contrast to the non-figurative window material, the purer roundel material resulted in the highest daylight transmission, which originates from similar glass thicknesses (Supplementary Table S4). This is seen in Fig. 9, where the transparency and brightness values of roundels IA1172 and IA4019 are higher compared with those of roundel AV8097. For this last roundel, it was noticed that despite its less pure material character, transparency values could be obtained that are comparable to those of roundels produced with much purer materials (roundels IA552 and IA1171). The reason for this can be ascribed to its lesser thickness (Supplementary Table S4). We remark that this roundel might originate from the Northern Low Countries. After the ‘Fall of Antwerp’ in 1585, the Low Countries were more or less divided into the Protestant Northern Netherlands and Catholic Southern Netherlands. In the sixteenth century, most roundels were executed on cylinder glass, but it is reported in the literature that especially in the Northern Netherlands, crown glass made a comeback by the end of the seventeenth century due to fabrication improvements resulting in larger crown diameters, thinner sheets and a better surface quality^5^.

## Methods

### Spectroscopic analysis

For each glass fragment, we recorded the transmittance spectrum T(λ) from 250 to 1250 nm. With this spectral range, we are able to study all defined optical measures—the UVAE, the colour values and the absorption bands in the visible part of the electromagnetic spectrum supplemented with the iron-absorption peak around 1100 nm. We obtained the intrinsic spectra (1-mm-thick sample) by dividing the calculated absorbance spectra by the measured sample thickness. To calculate the absorbance spectra, we used the formula of A(λ) =  − log_10_T(λ). For constructing the simulated spectra for a desired sample thickness, the procedure consisted of multiplying the intrinsic absorption spectrum with the simulated thickness and calculating the corresponding transmittance spectrum. For recording the spectra, a spectral broadband light source (Avalight-HAL + DHSBAL; Avantes) illuminated the sample. An optical spectrum analyser (AvaSpec-3648, AvaSpec-256-NIR1.7; Avantes) was used to measure the transmitted intensity as a function of the wavelength. The light that the light source emits, is focused to a spot size of 1.5 mm at the position of the glass fragment. All of the transmitted light is then captured via an integrating sphere (AvaSphere-30; Avantes). Spectra are recorded with a spectral resolution of 1.5 nm.

### Colour analysis

The colour values which we represent on the widely used CIELAB colour space (CIELab) are derived from the CIE 1931 XYZ colour values. For the first step, the measured transmission spectrum is multiplied with the colour matching functions and the spectrum of the CIE standard illuminant D65. We selected this illuminant because it is one of the standard models of average daylight. The integration of the three obtained spectra over the spectral region for which the human eye is colour sensitive (i.e., 380–780 nm) leads to three values: X, Y and Z. These values allow us in a final step to calculate the Lab colour values with the aid of the conversion formulas described in literature. Brightness value L is scaled between 0 (black) and 100 (white). The chromaticity green–red component is represented on the a axis (green in the negative direction and red in the positive direction), whereas the blue-yellow component is embodied by the b axis (blue in the negative direction and yellow in the positive direction)^34^.

### Calculation of the UVAE

We applied the methodology that our group previously defined to calculate the position of the UVAE for archaeological glasses^20^. For modern glass pieces this position corresponds to the wavelength at which the transmission is a certain percentage (10% or 50%) of the maximum transmission after the normalisation of the spectrum to a 5-mm-thick sample^33^. In the case of archaeological glasses, normalizing to a 5-mm-thick sample leads to too high of an absorption. In addition, the absorption bands of Fe^3+^ at 380, 420 and 440 nm hamper the determination of the UVAE position. Therefore, in this work, the UVAE is obtained by fitting the transmission with a linear curve in the ultraviolet spectral region and by determining the wavelength at which the fitted line goes to zero.

### Calculation of the colour difference ΔEab

Starting from two colours in the CIELAB colour space of (${L}_{1},{a}_{1},{b}_{1}$) and (${L}_{2},{a}_{2},{b}_{2}$), the CIE76 colour difference formula is defined as ^34^:

$${\Delta E}_{ab}=\sqrt{{({L}_{2}-{L}_{1})}^{2}+{({a}_{2}-{a}_{1})}^{2}+{({b}_{2}-{b}_{1})}^{2}}$$

### Chemical analysis

Scanning electron microscope—energy dispersive X-ray spectroscopy (SEM–EDX) measurements were performed with a JEOL 6300 scanning electron microscope equipped with an energy dispersive X-ray detector. With this instrument, secondary electron and backscattered electron images (BEI) of the embedded samples were registered. The spectra were collected for 200 s by using a 2 nA electron beam current, an accelerating voltage of 20 kV and a microscope magnification of 500. These parameters were found to be suitable for the quantitative analysis of glass without significant diffusion of sodium during the irradiation. The net intensities were calculated with the program AXIL (Analysis of X-rays by Iterative Least squares) and quantified by means of a standard less ZAF program ^39^.

## Conclusion

This paper is the result of a close collaboration between art historians and optical engineers in the field of heritage research, and it offers new insights into the interplay between used raw materials and fabrication technology in the economic flourishing period from the fifteenth to the seventeenth century in the (Southern) Low Countries. Starting from two well-diagnosed groups of excavated non-figurative glass window pieces, we researched the combined influence of the purity of the raw materials and the glass thickness obtained with either the crown or the cylinder method on the amount of transmitted light transmission. Earlier-performed macroscopic research unveiled a difference in the obtained glass thickness between both glass fabrication methods. First of all, the analysis of the recorded optical transmittance spectra demonstrated that the earlier-dated lozenge shaped glass is purer compared with the more recent quadrangular material, mainly due to the lower iron-impurity levels. Nonetheless, the study of the light transmission properties has shown that despite its better glass purity, the lozenge glass is less transparent compared with the more greenish quadrangular glass. Linking the observations of the spectroscopic research with earlier reported chemical data on a subset of samples allows us to conclude that the glass was produced in (a) local workshop(s) employing different fabrication technologies regarding the use of raw materials. The fact that the more recent material is made from less pure materials but finally leads to increased window transparency demonstrates that changing socio-economic realities prompted the ancient glassmakers to innovate. In a second case study, we assessed these research findings and studied six roundels originating from glass ateliers located in the (Southern) Low Countries. For this glass material, we also observed an evolution towards less pure materials in later periods. Despite this, more light was transmitted through the roundels compared with the non-figurative windows due to their lesser thickness. This can be seen as proof for their status as a luxury commodity.

The above-described insights on glass technology and the related daylight transmission indicate that as is the case in today’s world, societal and cultural needs strongly drove technological innovations, and glassworkers definitely succeeded in increasing daylight transmission as required by the Reformation and Counter-Reformation. This work can further contribute to historic research conducted in an attempt to unravel the relation between glazing choices and related social and economic encounters.

A similar research methodology can be applied in future research to validate the findings of this paper with materials from other sites, including the full range of existing glass pattern shapes.

## Supplementary Information

Supplementary Tables.
