# Supplementary material for: The interaction between daylight and fifteenth and sixteenth century glass windows from the Low Countries
Source: Sci Rep. 2021 Oct 29;11:21338. doi: 10.1038/s41598-021-00359-7 (PMC8556355; doi:10.1038/s41598-021-00359-7)
Supplement: Supplementary file 1 — Supplementary Tables. [file 41598_2021_359_MOESM1_ESM.pdf]

Supplementary Information

The interaction between daylight and 15th and 16th century glass windows from the Low Countries

Wendy Meulebroeck,[1,*] Karin Nys,[2] Mathilde Patin,[1] and Hugo Thienpont[1]

[1]Department of Applied Physics and Photonics, Brussels Photonics, Vrije Universiteit Brussel, Pleinlaan 2, 1050 Brussels, Belgium

[2]Department of Art Sciences and Archaeology, MARI Research Group, Vrije Universiteit Brussel, Pleinlaan 2, 1050 Brussels, Belgium

*Correspondence and requests for materials should be addressed to W.M. (email: wendy.meulebroeck@vub.be)

Supplementary Table S1. List of all non-figurative window samples analysed by SEM-EDX and optical spectroscopy. The table reports the optical parameters, colour coordinates, absorption at 1100 nm and UVAE. The total amount of iron content obtained by SEM-EDX is also reported. The last three columns show the Fe^{2+} data obtained by using the calibration curve of Ceglia and Fe^{3+} and $Fe^{2+}/\Sigma Fe_{tot}$ data obtained by mixing the information of SEM-EDX and optical analysis.

Sample	Pattern shape	Dating	Optical parameters					SEM-EDX	Derivated		
			L	a	b	A (1100nm)	UVAE (nm)	Fe_{tot} (wt.%)	Fe^{2+}	Fe^{3+}	$Fe^{2+}/\Sigma Fe_{tot}$
MIKA420_1	L.	15th c.	73.2	-2.1	2.1	0.39	336.1	0.62	0.33	0.29	0.53
MIKA421_1	L.	15th c.	79.6	-2.0	3.1	0.36	333.8	0.56	0.30	0.25	0.55
MIKA475_1	L.	15th c.	86.6	-3.1	2.1	0.28	335.8	0.64	0.24	0.40	0.37
MIKA475_2	L.	15th c.	91.4	-3.0	2.2	0.21	334.1		0.18		
MIKA488_1	L.	15th c.	71.3	-1.9	4.5	0.43	339.7	0.66	0.35	0.31	0.53
MIKA7_1	Q.	16th c.	83.1	-4.5	7.2	0.42	346.9	1.04	0.35	0.69	0.34
MIKA7_2	Q.	16th c.	87.6	-4.6	7.0	0.31	358.2		0.26		
MIKA67_1	Q.	16th c.	86.5	-4.3	5.9	0.35	343.7	1.09	0.29	0.79	0.27
MIKA67_2	Q.	16th c.	82.5	-4.1	6.2	0.40	343.9		0.33		
MIKA67_3	Q.	16th c.	85.1	-4.9	7.3	0.38	344.7		0.32		
MIKA67_4	Q.	16th c.	86.1	-5.3	7.0	0.40	342.9		0.33		
MIKA67_5	Q.	16th c.	87.7	-5.0	6.5	0.37	344.6		0.31		
MIKA79_1	Q.	16th c.	89.1	-4.4	6.3	0.33	342.8	1.12	0.28	0.84	0.25
MIKA79_3	Q.	16th c.	83.4	-5.1	7.1	0.47	345.5		0.39		
MIKA79_4	Q.	16th c.	86.3	-4.8	6.5	0.38	343.1		0.31		
MIKA79_5	Q.	16th c.	71.1	-4.1	7.7	0.58	345.5		0.48		
MIKA79_6	Q.	16th c.	82.2	-5.2	7.0	0.44	344.2		0.37		
MIKA82_1	Q.	16th c.	85.7	-5.2	6.2	0.36	344.0	1.09	0.30	0.79	0.27
MIKA82_2	Q.	16th c.	81.7	-3.7	8.3	0.39	344.5		0.32		

MIKA82_3	Q.	16th c.	83.8	-4.6	8.2	0.38	344.3		0.31		
MIKA82_4	Q.	16th c.	82.3	-4.4	7.6	0.39	344.8		0.32		
MIKA82_5	Q.	16th c.	83.4	-4.9	9.2	0.40	347.2		0.33		
MIKA82_6	Q.	16th c.	87.8	-4.6	7.0	0.35	343.4		0.29		
MIKA93_1	Q.	16th c.	82.2	-4.3	7.3	0.35	366.2	1.03	0.30	0.74	0.29
MIKA93_2	Q.	16th c.	85.4	-4.5	6.8	0.40	344.3		0.33		
MIKA94_1	Q.	16th c.	85.7	-4.9	8.5	0.35	351.4	1.01	0.30	0.71	0.29
MIKA94_2	Q.	16th c.	75.0	-3.8	8.9	0.48	347.8		0.40		
MIKA95_1	Q.	16th c.	84.9	-4.4	6.9	0.35	353.9	1.02	0.29	0.73	0.28
MIKA95_2	Q.	16th c.	81.7	-4.7	8.5	0.38	354.9		0.32		

Supplementary Table S2. Chemical composition of a subset of 11 non-figurative window samples analysed by SEM-EDX. Data are in wt.%.

Sample	Pattern shape	Dating	SiO_2	Al_2O_3	Fe_2O_3	TiO_2	MnO	Na_2O	K_2O	MgO	CaO	Cl	SO_3	P_2O_5	BaO	Total
MIKA420_1	L.	15th c.	59.7	4.07	0.62	0.16	0.80	1.31	6.90	2.83	20.44	1.02	0.01	1.61	0.27	99.8
MIKA421_1	L.	15th c.	61.3	2.89	0.56	0.13	0.56	2.09	4.61	2.71	21.23	1.71	0.01	1.89	0.10	99.8
MIKA475_1	L.	15th c.	60.3	4.06	0.64	0.21	0.74	0.81	7.95	3.47	19.43	0.58	0.01	1.29	0.28	99.8
MIKA488_1	L.	15th c.	60.1	4.08	0.66	0.15	0.84	1.38	6.29	2.75	20.51	0.96	0.01	1.76	0.28	99.8
MIKA7_1	Q.	16th c.	60.3	3.61	1.04	0.19	0.53	1.96	5.93	2.78	20.98	0.86	0.01	1.38	0.21	99.7
MIKA67_1	Q.	16th c.	60.1	3.74	1.09	0.20	0.52	1.95	5.85	2.78	21.08	0.92	0.01	1.29	0.20	99.7
MIKA79_1	Q.	16th c.	60.5	3.65	1.12	0.20	0.52	1.96	5.85	2.73	20.80	0.88	0.01	1.32	0.21	99.7
MIKA82_1	Q.	16th c.	60.2	3.58	1.09	0.19	0.54	2.03	6.03	2.84	20.64	1.00	0.01	1.37	0.20	99.7
MIKA93_1	Q.	16th c.	62.1	3.74	1.03	0.20	0.51	1.93	5.28	2.63	19.88	1.03	0.01	1.22	0.20	99.8
MIKA94_1	Q.	16th c.	60.2	3.69	1.01	0.20	0.52	1.95	5.96	2.78	21.01	1.15	0.01	1.10	0.19	99.8
MIKA95_1	Q.	16th c.	60.3	3.73	1.02	0.20	0.52	1.94	5.97	2.77	21.00	1.01	0.01	1.15	0.20	99.8

Supplementary Table S3. The table reports the optical parameters, colour coordinates and Transparency values for the spectra with simulated thicknesses of 3 mm and 2 mm for respectively the Lozenge and the quadrangular non-figurative window pieces.

Sample	Pattern shape	Dating	Simulated thickness (mm)	L	a	b	Transparency (%)
MIKA420_1	L.	15th c.	3	36.7	-3.7	3.5	8.5
MIKA421_1	L.	15th c.	3	48.9	-3.9	6.2	15.0
MIKA475_1	L.	15th c.	3	64.4	-7.2	4.8	29.0
MIKA475_2	L.	15th c.	3	76.0	-7.5	5.5	43.9
MIKA488_1	L.	15th c.	3	33.4	-2.8	7.3	7.0
MIKA7_1	Q.	16th c.	2	68.6	-7.3	11.9	31.8
MIKA7_2	Q.	16th c.	2	76.6	-7.7	12.1	41.8
MIKA67_1	Q.	16th c.	2	74.6	-7.4	10.0	39.4
MIKA67_2	Q.	16th c.	2	67.7	-6.7	10.1	31.0
MIKA67_3	Q.	16th c.	2	72.1	-8.2	12.2	35.0
MIKA67_4	Q.	16th c.	2	73.9	-8.8	11.8	37.1
MIKA67_5	Q.	16th c.	2	76.8	-8.6	11.2	41.3
MIKA79_1	Q.	16th c.	2	79.2	-7.6	11.1	45.3
MIKA79_3	Q.	16th c.	2	69.3	-8.2	11.8	31.5
MIKA79_4	Q.	16th c.	2	74.2	-8.0	11.1	38.5
MIKA79_5	Q.	16th c.	2	49.5	-5.6	11.0	14.3
MIKA79_6	Q.	16th c.	2	67.2	-8.4	11.5	29.3
MIKA82_1	Q.	16th c.	2	73.2	-8.8	10.5	37.3
MIKA82_2	Q.	16th c.	2	66.4	-5.8	13.5	29.7

MIKA82_3	Q.	16th c.	2	69.8	-7.3	13.5	33.1
MIKA82_4	Q.	16th c.	2	67.3	-6.9	12.3	30.2
MIKA82_5	Q.	16th c.	2	69.2	-7.7	15.1	31.8
MIKA82_6	Q.	16th c.	2	76.9	-7.7	12.0	41.9
MIKA93_1	Q.	16th c.	2	67.2	-6.8	11.8	29.9
MIKA93_2	Q.	16th c.	2	72.7	-7.5	11.4	36.5
MIKA94_1	Q.	16th c.	2	73.2	-7.9	14.3	36.9
MIKA94_2	Q.	16th c.	2	55.4	-5.4	13.3	19.2
MIKA95_1	Q.	16th c.	2	71.8	-7.3	11.6	35.7
MIKA95_2	Q.	16th c.	2	66.3	-7.4	13.7	29.3

Supplementary Table S4. The table reports the optical parameters, colour coordinates and Transparency values for the six studied roundels.

Roundel No.	Collection *	Inv. No.	Dating	Origin	Measurement no.	Thickness (mm)	Glass parameters (purity)					Glass parameters (light transmission)			
							L	a	b	A (1100 nm)	UVAE (nm)	L	a	b	Transparency (%)
1	RMAH	IA552	1st half 16th c.	Southern Low Countries, Brussels	IA552_1	2.0	92.9	-3.3	2.0	0.31	335.1	83.8	-5.9	3.5	56.1
					IA552_2	1.8	93.5	-3.6	1.8	0.31	334.3	85.7	-6.0	3.0	59.9
					IA552_3	1.8	90.5	-2.5	3.1	0.30	331.9	80.5	-4.1	5.2	52.0
2	MAS	AV1172	1st half 16th c.	Southern Low Countries, Antwerp	AV1172_1	1.5	97.1	-2.2	3.9	0.14	341.8	92.8	-3.0	5.5	76.9
					AV1172_2	1.3	97.1	-2.4	4.2	0.15	342.3	93.2	-2.9	5.2	78.2
					AV1172_3	1.2	96.6	-2.0	4.4	0.14	342.3	92.8	-2.4	5.3	77.8
					AV1172_4	1.2	95.9	-2.4	5.7	0.15	343.7	91.9	-2.9	6.8	75.3
3	RMAH	IA4019	Mid 16th. c.	Southern Low Countries, Leuven	IA4019_1	1.7	95.3	-1.8	4.3	0.15	338.0	89.4	-2.7	6.8	70.3
					IA4019_2	1.8	95.2	-2.0	3.7	0.15	334.4	88.7	-3.3	6.1	68.7
					IA4019_3	1.8	95.3	-1.9	3.7	0.15	338.4	88.6	-3.1	6.3	68.4
					IA4019_4	1.9	95.8	-1.8	3.8	0.14	335.2	89.2	-3.2	6.9	69.5
					IA4019_5	1.8	95.3	-1.7	3.8	0.14	334.0	88.9	-2.7	6.2	69.4
					IA4019_6	2.0	95.5	-1.6	3.1	0.14	330.3	88.5	-3.0	5.6	68.4
4	MAS	AV1171	2nd half 16th. c.	Southern Low Countries, Antwerp	AV1171_1	1.9	93.5	-3.8	5.4	0.26	345.4	85.0	-6.6	9.4	57.7
					AV1171_2	1.9	93.4	-3.8	5.6	0.26	346.0	84.8	-6.5	9.7	57.3
					AV1171_3	2.0	92.5	-3.2	7.1	0.25	343.3	82.6	-5.5	12.7	53.7
					AV1171_4	2.0	92.4	-3.5	6.2	0.26	344.3	82.5	-6.1	10.9	53.4

5	RMAH	IA4039	2nd quarter 17th c.	Southern Low Countries, Leuven	IA4039_1	1.5	83.0	-4.5	6.4	0.36	353.0	72.9	-6.0	8.5	41.3
					IA4039_2	1.8	83.9	-4.6	7.6	0.35	351.2	70.7	-6.8	11.4	37.5
					IA4039_3	1.8	85.9	-5.1	5.9	0.33	348.1	73.3	-7.9	9.0	40.9
					IA4039_4	2.1	84.7	-4.4	4.8	0.34	340.6	68.5	-7.4	7.9	34.9
					IA4039_5	2.1	85.0	-4.9	5.6	0.33	324.9	68.4	-8.3	9.5	34.3
					IA4039_6	2.1	84.7	-5.1	5.7	0.34	351.0	67.7	-8.6	9.7	33.1
					IA4039_7	2.0	81.4	-4.4	9.9	0.36	349.1	63.9	-6.5	15.4	29.2
					IA4039_8	1.4	85.0	-4.8	6.3	0.35	355.9	77.0	-6.2	8.1	47.3
6	MAS	AV8097	17th c.	Low Countries	AV8097_1	0.8	88.3	-6.0	11.1	0.45	368.3	88.0	-4.7	8.7	65.0
					AV8097_2	0.9	88.5	-5.9	10.5	0.43	366.9	86.5	-5.3	9.5	61.5
					AV8097_3	0.9	81.4	-5.0	11.7	0.49	368.9	80.0	-4.6	10.7	50.4
					AV8097_4	0.8	78.8	-4.1	11.2	0.49	367.9	80.6	-3.3	8.9	52.8

* RMAH – Art & History Museum (Brussels), MAS-Vleeshuis museum (Antwerp)
